# Supplementary material for: The impact of regional astrocyte interferon-γ signaling during chronic autoimmunity: a novel role for the immunoproteasome
Source: J Neuroinflammation. 2020 Jun 12;17:184. doi: 10.1186/s12974-020-01861-x (PMC7291495; doi:10.1186/s12974-020-01861-x)
Supplement: Supplementary file 2 — Additional file 2: Figure S2. IFNγ-regulated expression of the constitutive proteasome in regional human astrocytes. Human brainstem and spinal cord astrocytes were stimulated with 10 ng/ml IFNγ for 0, 6, 12, 24, or 48 h and RNA was collected and analyzed for transcript levels of PSMB5, PSMB6, and PSMB7 by qRT-PCR, normalized to copies of GAPDH. Data represent the mean ± SEM from 3 independent experiments. **P < 0.01, ***P < 0.001 between regions by 2-way ANOVA. [file 12974_2020_1861_MOESM2_ESM.pdf]

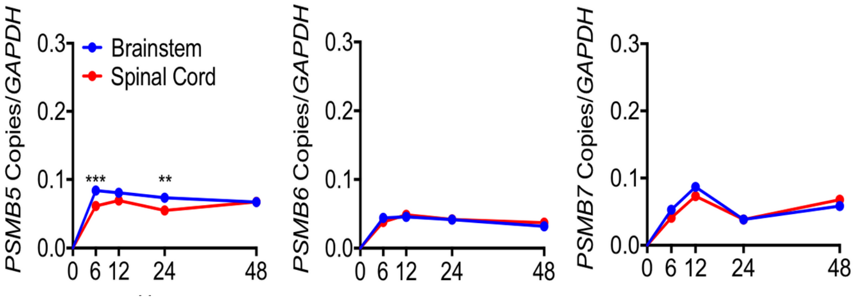

Figure S2: IFNγ-regulated expression of the constitutive proteasome in regional human astrocytes. Human brainstem and spinal cord astrocytes were stimulated with 10 ng/ml IFNγ for 0, 6, 12, 24, or 48 h and RNA was collected and analyzed for transcript levels of *PSMB5*, *PSMB6*, and *PSMB7* by qRT-PCR, normalized to copies of *GAPDH*. Data represent the mean ± SEM from 3 independent experiments. $^{**}P < 0.01$, $^{***}P < 0.001$ between regions by 2-way ANOVA.
