# Supplementary material for: The impact of regional astrocyte interferon-γ signaling during chronic autoimmunity: a novel role for the immunoproteasome
Source: J Neuroinflammation. 2020 Jun 12;17:184. doi: 10.1186/s12974-020-01861-x (PMC7291495; doi:10.1186/s12974-020-01861-x)
Supplement: Supplementary file 3 — Additional file 3: Figure S3. Regional inflammation and iP expression in Ifngr1fl/flTie2-Cre+ mice. EAE was induced in Ifngr1fl/flTie2-Cre+ mice (n = 5) and (A) EAE clinical course was monitored. Following 30 days post-immunization, mice were perfused and the CNS was removed and cryopreserved for IHC analysis. White matter tracts of the brain stem and lumbar spinal cord were imaged using confocal microscopy at 20x magnification. (B) Tissue sections were labeled for MBP (red) and nuclei were counterstained with DAPI (blue). (D) Lesion area was quantified using ImageJ software. (C) Tissue sections were labeled for LMP2 (green), GFAP (red), and nuclei were counterstained with DAPI (blue). (F) Total LMP2 area and LMP2 colocalized with GFAP were analyzed. *P < 0.05 between regions by 2-tailed Student’s t test. [file 12974_2020_1861_MOESM3_ESM.pdf]

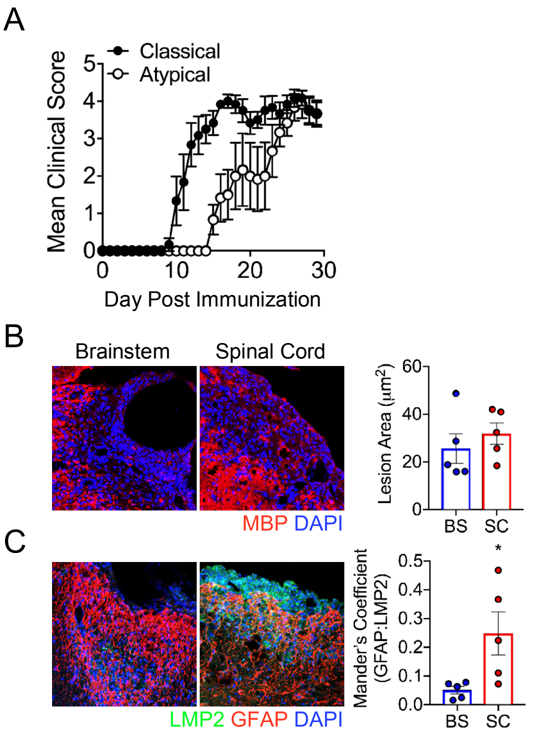

Figure S3: Regional inflammation and iP expression in *Ifngr1*$^{fl/fl}$*Tie2*-Cre^{+} mice. EAE was induced in *Ifngr1*$^{fl/fl}$*Tie2*-Cre^{+} mice (n = 5) and **(A)** EAE clinical course was monitored. Following 30 days post-immunization, mice were perfused and the CNS was removed and cryopreserved for IHC analysis. White matter tracts of the brain stem and lumbar spinal cord were imaged using confocal microscopy at 20x magnification. (**B**) Tissue sections were labeled for MBP (red) and nuclei were counterstained with DAPI (blue). (**D**) Lesion area was quantified using ImageJ software. **(C)** Tissue sections were labeled for LMP2 (green), GFAP (red), and nuclei were counterstained with DAPI (blue). **(F)** Total LMP2 area and LMP2 colocalized with GFAP were analyzed. *P < 0.05 between regions by 2-tailed Student's *t* test.
