# Supplementary material for: The impact of regional astrocyte interferon-γ signaling during chronic autoimmunity: a novel role for the immunoproteasome
Source: J Neuroinflammation. 2020 Jun 12;17:184. doi: 10.1186/s12974-020-01861-x (PMC7291495; doi:10.1186/s12974-020-01861-x)
Supplement: Supplementary file 4 — Additional file 4: Figure S4. IFNGR1 deletion in astrocytes of Ifngr1fl/flGfap-Cre+ mice. (A) IHC detection of astrocyte marker GFAP (red) or (B) Iba1 (red) and IFNGR1 (green) in the ventral spinal cords of Ifngr1fl/fl and Ifngr1fl/flGfap-Cre+ mice at day 25 post-EAE induction. Nuclei are shown in blue. Images are representative of at least 4-20x images for each of 7 independent mice per genotype. Colocalization is quantified by Mean Mander’s Coefficient using ImageJ software. Data points are representative of individual mice. **P < 0.01 between genotypes by 2-tailed Student’s t test. [file 12974_2020_1861_MOESM4_ESM.pdf]

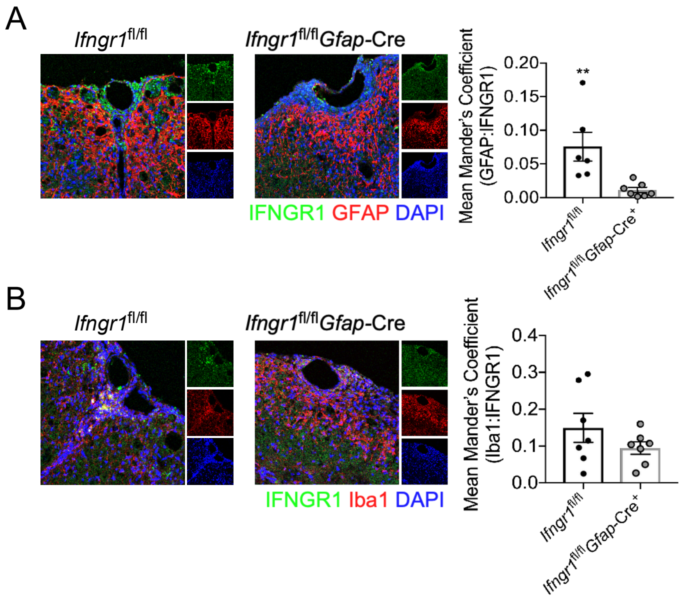

Figure S4: IFNGR1 deletion in astrocytes of *Ifngr1*$^{fl/fl}$*Gfap*-Cre^{+} mice. (A) IHC detection of astrocyte marker GFAP (red) or **(B)** Iba1 (red) and IFNGR1 (green) in the ventral spinal cords of *Ifngr1*$^{fl/fl}$ and *Ifngr1*$^{fl/fl}$*Gfap*-Cre^{+} mice at day 25 post-EAE induction. Nuclei are shown in blue. Images are representative of at least 4-20x images for each of 7 independent mice per genotype. Colocalization is quantified by Mean Mander's Coefficient using ImageJ software. Data points are representative of individual mice. **P < 0.01 between genotypes by 2-tailed Student's t test.
