# Supplementary material for: The impact of regional astrocyte interferon-γ signaling during chronic autoimmunity: a novel role for the immunoproteasome
Source: J Neuroinflammation. 2020 Jun 12;17:184. doi: 10.1186/s12974-020-01861-x (PMC7291495; doi:10.1186/s12974-020-01861-x)
Supplement: Supplementary file 5 — Additional file 5: Figure S5. LMP2 is not reduced in microglia/monocytes in Ifngr1fl/flGfap-Cre+ mice. IHC detection of Iba1 (red) and LMP2 (green) in the ventral spinal cords of Ifngr1fl/fl and Ifngr1fl/flGfap-Cre+ mice at day 25 post-EAE induction. Nuclei are shown in blue. Colocalization is quantified by Mander’s coefficient using ImageJ software. Data points are representative of individual mice. Data represent the mean ± SEM combined from 2 independent experiments and were analyzed by 2-tailed Student’s t test. [file 12974_2020_1861_MOESM5_ESM.pdf]

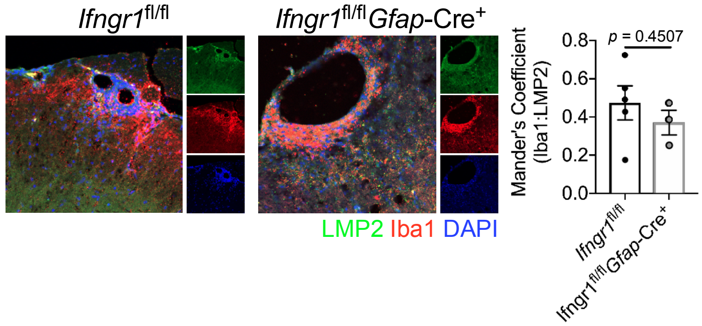

Figure S5: LMP2 is not reduced in microglia/monocytes in *Ifngr1*$^{fl/fl}$*Gfap*-Cre^{+} mice. IHC detection of Iba1 (red) and LMP2 (green) in the ventral spinal cords of *Ifngr1*$^{fl/fl}$ and *Ifngr1*$^{fl/fl}$*Gfap*-Cre^{+} mice at day 25 post-EAE induction. Nuclei are shown in blue. Colocalization is quantified by Mander's coefficient using ImageJ software. Data points are representative of individual mice. Data represent the mean ± SEM combined from 2 independent experiments and were analyzed by 2-tailed Student's *t* test.
